# Design, Implementation and Power Analysis of Pervasive Adaptive Resourceful Smart Lighting and Alerting Devices in Developing Countries Supporting Incandescent and LED Light Bulbs

**DOI:** 10.3390/s19092032

**Published:** 2019-04-30

**Authors:** Preethi Sambandam Raju, Murugan Mahalingam, Revathi Arumugam Rajendran

**Affiliations:** 1 Department of Electronics and Communication Engineering, SRM Valliammai Engineering College, Kattankulathur 603203, India; vp@valliammai.co.in; 2 Research Scholar, Department of Information and Communication Engineering, Anna University, Chennai 600025, India; 3 Department of Information Technology, SRM Valliammai Engineering College, Kattankulathur 603203, India; revathiar.it@valliammai.co.in

**Keywords:** lighting control, Arduino, light dimmer module, energy saving ratio, power analysis, energy consumption ratio

## Abstract

Nowadays, there is an increasing demand for energy saving techniques in residential, industrial, institutional, clinical and other multipurpose indoor and outdoor applications. Lights play an ubiquitous role around the Earth in all types of structures and outdoor surroundings. Hence, the authors propose a universal lighting control device—named Pervasive Adaptive Resourceful Smart Lighting and Alerting Device—accomplished mainly by the use of Arduino UNO R3. The Pervasive Adaptive Resourceful Smart Lighting and Alerting Device works in two modes, namely, light control and alert, by deploying the perceptive light automation and perceptive light automation with buzzer activation algorithms, respectively. The contributions of the paper are: a common lighting control solution for both incandescent and light emitting diode light bulbs for all indoor and outdoor environments. A profound power consumption analysis, and investigation of the proposed device by estimating the Energy Consumption Ratio (ECR) and Relative Energy Saving Ratio (RESR) through the real time deployment in diverse circumstances with 60 W incandescent, 8 W and 0.5 W LED light bulbs is executed. In addition to the evaluation of RESR and ECR characteristics the power consumption of light bulbs in terms of scalable conditions of number of light bulbs is also analyzed. The proposed model is proved to work efficiently for both incandescent and LED light bulbs.

## 1. Introduction

Smart automation is used primarily for patient monitoring [1], activity recognition [2,3] and energy management [4,5]. Though smart automation is now made possible with the enormous growth in Internet of Things (IoT); it faces three main difficulties, namely device heterogeneity, inconsistency and conflicting decisions [6]. Heterogeneity is mainly due to the varied volume and format of data from different sensors deployed for sensing the environment. These diverse data become incomparable and unpredictable, leading to contradictions when predicting the environment conditions to proceed further in a sequence of automated actions. Thus, heterogenous data causes inconsistency which eventually ends up in conflicting decisions. The electronic accessories deployed can be categorized into four types, such as basic utilities, comfort gadgets, pleasure-providing appliances and heavy equipment. Basic utilities include various forms of lights and fans that are significant tools leading to undesirable waste of power. Comfort gadgets comprise the newly designed apparatus used for relaxed living such as heating (heaters, induction stoves, kettles), cooling (refrigerators), lifts/escalators, computers, printers, air conditioning and washing (clothes and vessels). Pleasure-providing appliances, mainly utilized for recreation, include televisions, radios and phones. Heavy equipment comprises the massive machineries used in industrial units. 

Light bulbs prevailing in the small rooms of many storied buildings and in outdoor environments like streets, playgrounds, parks or gardens. The projected model predominantly controls only the lights, categorized under the basic devices as these are found in households of any kind, industries, hospitals and institutions, irrespective of their location across the globe. The control of essential and all-time use equipment leads to a substantial power savings. In developing countries like India, the use of incandescent bulbs is still prevalent [7], so the authors have come up with a device that could control both incandescent and LED light bulbs to help developing countries minimize the power consumption. Further, from the cited analysis [7] it is found that 60 W and 100 W are the most popular incandescent bulbs used, so in experiments the authors use a 60 W incandescent light bulb which has 800 Lumens. Similarly, the authors chose a 8 W LED bulb which has 800 Lumens. For testing the performance of the proposed device with low power consuming appliances, a light bulb of 0.5 W is also used. 

**Figure 1:**
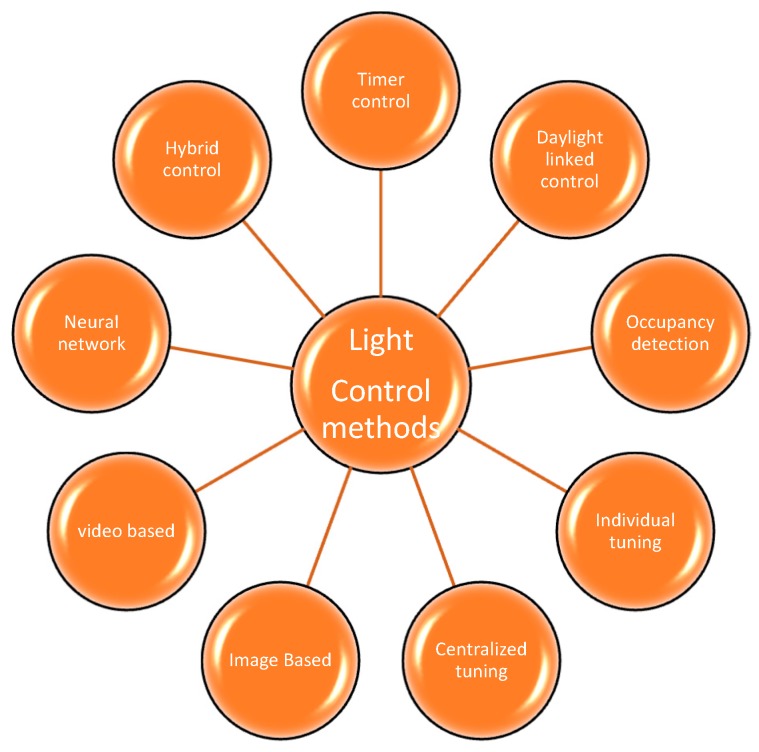
A survey of different light control methods.

Based on the previous work in lighting control by different authors around the world, light bulb control was found to be achieved by nine methods: timer control, daylight-linked control, occupancy detection, individual tuning, centralized tuning, image-based, video analytics, neural network-based control and hybrid control (Figure 1). Timer-based light control system were designed to set the status of light bulbs based on a preprogrammed time [8]. Daylight-linked control systems [9,10,11] were devised to operate by evaluating the daylight illuminance. The system design involves daylight availability, intensity evaluation and the assessment of daylight values. Based on the illuminance of daylight, dimming of light bulbs is performed. The two aspects required in daylight-linked systems are photosensors and dimming of light bulbs. Dimming affects the power quality of light bulbs [12]. In daylight intensity estimation, photosensors play a major role. The problems faced by photosensors are the distribution of daylight and artificial lighting in the space in which they are placed; the absence of explicit guidelines for the setting, positioning and commissioning of the photosensor; the spectral composition of lighting; the ambient light intensity and the field of view [13]. A decision-making technique [14] capable of identifying the best photosensor placement with variable field of view was analyzed based on three criteria. The three criteria were lighting levels between ceiling and working plane, lighting capability and energy savings. In a daylight control system, various parameters [15] were calculated and the energy savings were proved to vary in different places and under different conditions. Occupancy detection control is modeled by checking the motion or presence to trigger the light bulbs. This can also be implemented with vacancy sensors. Various occupation detection methodologies were presented in a review of occupancy sensing methodologies [16,17]. In conditions where occupants stay active for all time, dimming is found to save more energy [18]. Individual tuning [19,20] is adjustment of the light intensity level to the user’s comfort which is categorized as personal tuning. This control technique was selected by single users. In centralized tuning or institutional tuning [21,22] a cluster of light bulbs is controlled by location or building- specific conditions. In image-based control the luminance was calculated by the image captured by a low cost camera [23], CMOS low resolution camera [24] or CCD camera [25]. In video analytics-based control [26], lights in places like underground garages were controlled by video analytics. Artificial neural networks, deep learning techniques and machine learning are the most recent methods deployed to control the light [27,28]. When one or more of the above discussed techniques [29,30,31] are combined, they form a hybrid control technique. 

Sung [6] proposed a smart LED lighting system with the use of a wireless light dimmer, a multimeter, IR modules, a touch pad as a human computer interface and a self-adaptive weighted data fusion algorithm. A smart home energy management was built with a low power microprocessor using light, humidity and temperature sensors along with a disjoint multipath routing protocol, Zigbee and IEEE 802.15.4 [31]. A LED lighting system for green buildings was presented with CC2530 for Zigbee management, MSP430 for smoothing control of LED panel, and a dimmable LPF-40D-42 MeanWell commercial driver [32]. Lighting control for multipurpose outdoor environments [33] addressed efficiency at the component level and optimized the performance of LED drivers at the system level, by defining the control strategy and associated hardware infrastructure. Pellegrino et al. developed a lighting control system for historical buildings using daylight harvesting, absence control, Thermoken SR-MDS solar sensors, Eltako switches, Eltako actuators and ST-Microelectronics smart plugs [34]. An automation system for controlling streetlights [28], was designed with an Arduino microcontroller, a light dependent resistor (LDR) and infrared-sensors. In this work, the number of vehicles crossing the street lights was also counted. Xu et al. analyzed the energy saving potential of various lighting control strategies [35]. A wireless mesh networked lighting system [25], was considered with daylight and occupancy adaptive control through multiple sensor-equipped luminaires and a central controller. Building-in-Briefcase (BiB) [17] is a portable sensor network platform for continuous monitoring of building occupancy and the environment. A lighting automatic control system [36] made adjustments in lighting intensity, incorporating user illumination requirements based on their activities and achieved changes based on external lighting. An architecture [21], centralizing the control of lighting was merged with numerous artificial intelligence methods.

After a study of the relevant literature, the task is to provide one common solution for both indoor and outdoor lighting solutions. Also, the proposed model is suitable for developing countries that have both incandescent and light emitting diode bulbs in use. The Pervasive Adaptive Resourceful Smart Lighting and Alerting Device (PARSLAD) has an alarm system in the operation of the alert mode, so, monitoring and control can be achieved with one device. The alert mode is for future development with deep learning and video surveillance in the next upcoming work planned by the authors. Further, this is an initial prototype designed and tested on a small scale for future expansion by the authors to control surveillance and light bulbs in a city. In future, the enhanced PARSLAD system will focus on control of all the light bulbs (outdoor and indoor) along with cameras for monitoring an entire city in India. A short analysis of the proposed system in comparison with existing methods is given in Table 1.

The foremost contributions of the paper are summarized as follows: a universal adaptive and efficient lighting control device implemented through a perceptive light automation (PLA) algorithm and perceptive light automation with buzzer activation (PLABA) for smart supervision of both LED and incandescent light bulbs. Next, operation of PARSLAD in two modes of operation: light control (LC) and alert (AL) is studied. Compared to the available literature on lighting solution, the authors’ work starts with the suggestion of a unique device for controlling light along with protection actions, followed by analysis of power, energy consumption ratio (ECR) and relative energy saving ratio (RESR) of a PARSLAD prototype under diverse conditions and numerous situations. Lastly, our future work on a Raspberry Pi-based lighting control solution with surveillance features is indicated.

The remainder of the paper is organized as follows: Section 2 deals with the materials and methods for the design and implementation of PARSLAD. Section 3 provides details of the power analysis of PARSLAD performance in three scenarios. Section 4 discusses the energy consumption of for different number of bulbs in varied scenarios along with the characteristics of ECR, RESR and ES and cost analysis. Section 5 concludes the paper along with future work.

## 2. Materials and Methods

Pervasive Adaptive Resourceful Smart Lighting Device (PARSLAD) uses perceptive light automation (PLA) and perceptive light automation with buzzer activation (PLABA) algorithms. The device comprises the components tabulated in Table 2. 

**Figure 2:**
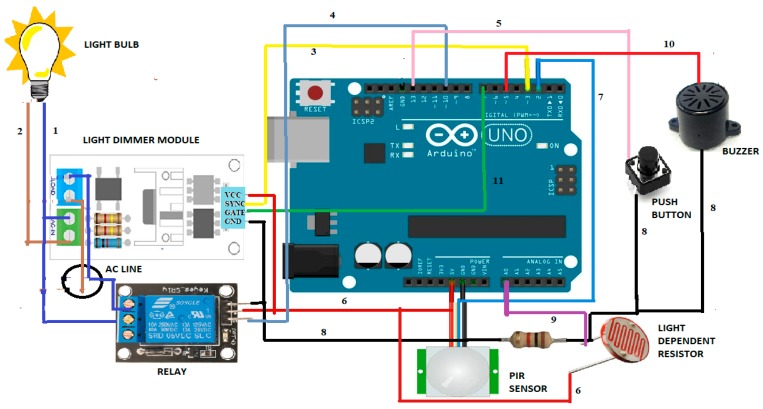
PARSLAD architecture. 1,2—Indicates AC line; 3—SYNC of light dimmer and 3^rd^ pin of the Arduino board; 4—signal pin of relay and 10^th^ pin of the Arduino; 5—push button and 13^th^ pin of the Arduino; 6—power supply to 5 V V_CC_ of the Arduino; 7—output of PiR to the 2^nd^ pin of the Arduino; 8—ground pin to GND of the Arduino; 9—LDR to A0 of the Arduino; 10—Buzzer to 5^th^ of the Arduino; 11—Gate of light dimmer to 7^th^ of the Arduino.

**Figure 3:**
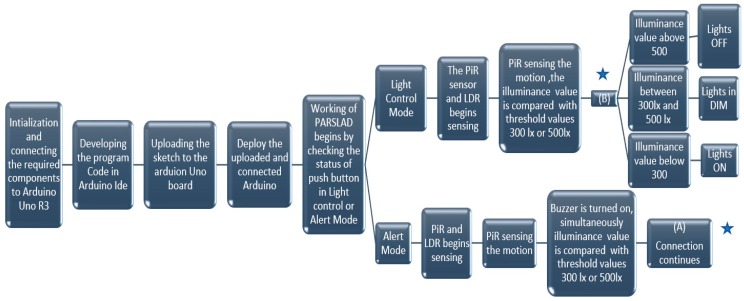
PARSLAD methodology. ★ Indicates the connection flow between A and B.

An Arduino UNO [37,38] R3 board is the heart of the circuit. The light dimmer module has AC input and an AC load port along with Vcc, ground, gate and SYNC pin. In certain models, SYNC pins are denoted as ZC or PWM pin. The gate and SYNC of the dimmer light module are connected to the 7^th^ and 3^rd^ pins of the Arduino Uno, respectively. The light dimmer works based on the zero crossing detector and opening the triac after a delay that gives a dimming effect to light bulbs [39]. The relay has six pins, with the signal pin connected to the 10^th^ pin of Arduino Uno board and C and NO pin given to the control device. The PiR sensor has three pins and the output pin is connected to the 2^nd^ pin of the Arduino Uno board. A LDR is connected between the power and the A0 pin. A push button and the buzzer are assigned to the 13^th^ and 5^th^ pin of the Arduino. All the components are supplied power through a 5 V pin and are grounded to the GND pin. Figure 2 and Figure 3 illustrate implementation and methodology of PARSLAD with an Arduino board, respectively.

Two modes of operation are possible in PARSLAD– light control (LC) and alert (AL) based on the condition of the push button. In LC mode of operation, merely the lighting control works driving PLA whereas, in the AL mode, lighting control along with buzzer works to indicate intervention by stimulating with the perceptive light automation with buzzer activation algorithm. PARSLAD is operated in the LC mode for places like restrooms, small homes, offices, hospitals, institutions, all time public gathering places, roads, gardens, and parks that have frequent human intervention. AL mode functions when monitoring is required like for high security places, trespassing in restricted environments, lonely places, locked homes and offices itemized as spots with rare human intervention. Moreover the use of modes is based on the device user’s choice. A comparison of two modes based on the specific functionality is tabulated in Table 3.

The light dependent resistor (LDR) is a variable resistor which reading fluctuates according to the incident light intensity. The conversion of LDR readings into illuminance values (lx) is performed in the PARSLAD device. The resistance value of the LDR declines with increasing light intensity. The LDR reading obtained t Arduino pin A0 is the LDR voltage referenced to 5 V. In the Arduino, 5 V is fragmented into 1024 values., so the LDR voltage at the present illumination is obtained by Equation (1) and the illuminance by Equation (2). The recommended illuminance value [40] for most types of activity lies between 300 lx and 500 lx. The threshold illuminance values are set as 300 lx and 500 lx for light operations:

(1) $$V_{LDR}=LDRreading\times \frac{5}{1024}\;V$$

(2) $$IL=\frac{250}{V_{LDR}}-50\;\mathrm{lx}$$

### 2.1. Perceptive Light Automation Algorithm for Light Control Mode

PLA algorithm functions with the light bulbs in three states—ON, DIM and OFF—as tabulated in Table 4 by checking two conditions:

(1) PIR sensor detection of intervention
(2) Daylight detector sensing the intensity of daylight in the deployment site.

On true conditions of the first case with illuminance value less than 300 lx, light bulbs will be turned on while for factual condition of the first instance with illuminance value greater than 300 lx but less than 500 lx the lights will turn to a dim state. False of the first condition will not invoke the PLA algorithm. Thus, a reduction in unwanted energy consumption is possible when there is an essential daylight distribution and when no intrusion occurs around the adjacent zone of the bulb.

### 2.2. Perceptive Light Automation with Buzzer Activation Algorithm for Alert Mode

**Figure 4:**
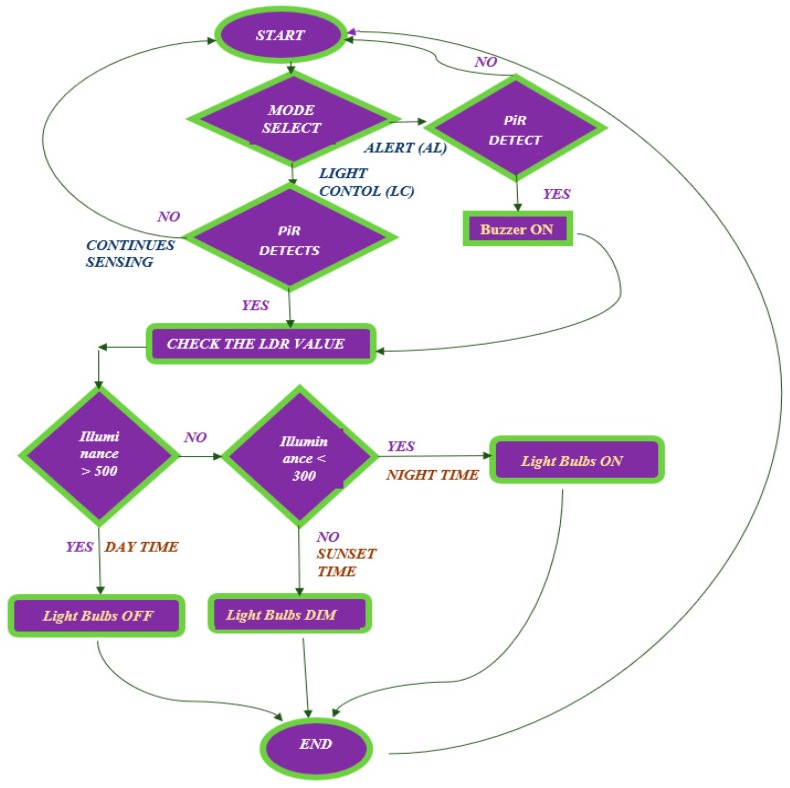
Flowchart of PARSLAD.

PARSLAD uses the PLABA algorithm for the AL mode, operating the light bulb in three states and the buzzer for alarms in two states. The operation of the light is similar to that of the PLA algorithm but, in PLABA, the buzzer is initiated to the ALARM ON state in ON and DIM states of lights (Table 5). Specifically, the buzzer sound triggers at detection of any intervention. Therefore, this alert with buzzer helps becoming aware of any sort of snooping in highly protected places. The AL mode of PARSLAD monitors homes or offices when the usual inhabitants are unavailable. This mode empowers the PARSLAD as a surveillance system avoiding the need for a separate monitoring device and thus lessens the cost and power consumption of a separate surveillance system. Figure 4 provides the full overview of PARSLAD with PLA and PLABA in flowchart form. 

## 3. Results

### 3.1. Energy Assessment Parameters Introduction and Formulation

For the purpose of investigation, four energy estimation parameters are used in this paper. They are energy consumption ratio of base scenario contrary to smart scenario (ECR_BS_), energy consumption ratio of manually controlled scenario counter to smart scenario (ECR_MS_), relative energy saving ratio of base scenario contrary to smart scenario (RESR_BS_) and relative energy saving ratio of manually controlled scenario counter to smart scenario (RESR_MS_). 

ECR_BS_ and ECR_MS_ are the ratios of energy consumed for a particular strategy by PARSLAD deployment to the energy consumed in the base and manually controlled scenario, respectively. 

RESR_BS_ and RESR_MS_ is the ratio of energy saved through use of PARSLAD device in a specific condition relative to the energy consumed in base and manually controlled scenario, respectively under the same specific conditions:

(3) $${\mathrm{ECR}}_{\mathrm{BS}}=\frac{E_{S}}{E_{B}}\times 100\%$$

(4) $${\mathrm{ECR}}_{\mathrm{MS}}=\frac{E_{s}}{E_{M}}\times 100\%,$$

(5) $${\mathrm{RESR}}_{\mathrm{BS}}=\frac{E_{B}-E_{s}}{E_{B}}\times 100\%$$

(6) $${\mathrm{RESR}}_{\mathrm{MS}}=\frac{E_{M}-E_{s}}{E_{M}}\times 100\%,$$

where, E_B_ is the energy expended in base Scenario, E_M_ is the energy consumed in the manually controlled scenario and Es is the energy spent in a smart scenario in KWh.

### 3.2. Power Analysis of PARSLAD and the Light Bulbs

An Absolute Native Electronics OLED USB detector which has built-in measurement devices like a voltmeter, ammeter, power capacity tester meter is used for the power analysis of devices. The range of voltage and current is 3.5–9 V and 0–3.3 A, respectively.

#### 3.2.1. Power Analysis of Varied Scenarios of Pervasive Adaptive Resourceful Smart Lighting and Alerting Device (PARSLAD)

Details of the analysis of ten varied scenarios of the PARSLAD and with different possible combinations of daylight distribution and residents’ auxiliary are provided in Table 6. The table also details the power, annual energy consumed, annual cost in both domestic and commercial conditions. Annual Kilowatt/hour values are calculated assuming that the device operates for 12 h a day for a year, resulting in 4380 operating hours annually. Time of operation is considered 12 h (5 pm to 5 am) in a day because inclusive of all seasons, the sunrise is between 5 am and 6 am while sunset is between 5 pm and 6pm in India. The cost for the annual operation is calculated in two aspects- domestic (Dom) and commercial (Com). In India, the electricity cost differs for Dom and Com use. In both Dom and Com use, base rates are fixed based on the user consumption, so for commercial use [41], the mean cost is taken as 0.12$ (8 Rupees and 30 Paise) per unit (1 KWh) and for domestic use [42], the mean cost is taken as 0.059$ (4 Rupees and 5 Paise) per unit (1 KWh). For commercial electricity rates, the mean value considering all the distributors is calculated. For the domestic electricity rate, the mean value for cost of >500 consumption units is calculated as more homes consume more than 500 units. From Table 6, it could be noted that the annual Dom and Com consumption cost of the proposed device is very cheap and could be used in developing countries and interior village people too for effective power savings.

#### 3.2.2. Annual Power Analysis and Usage Cost of 60 W Incandescent, 8 W LED and 0.5 W LED Light Bulbs Controlled or Not by PARSLAD

Table 7 charts the power analysis for the 60 W incandescent, 8 W LED and 0.5 W LED bulbs for two major conditions – deprived of PARSLAD and controlled by PARSLAD. Time of operation, domestic and commercial cost are fixed as stated above in Section 3.2.1.

### 3.3. Comparison of PARSLAD Performance in Three Scenarios

On a normal sunny day a real time deployment was carried out at 12.9898° N, 80.1434° E latitude and longitude location on 17^th^ August 2018. For the purpose of calculating the efficiency of the PARSLAD device on the power consumption of a 60 Watts incandescent bulb inside a room, the sensor is placed near the window where there is a possibility of penetration of sunrays in ON state from 5:00 pm to 9:00 pm was noted. Examination of sunset timing demonstrates the efficiency of PARSLAD, as the value of daylight changes drastically and changes the LDR values resulting in variations of illumination. Similarly, the existence of intervention is in two time slots one; between 5:00 pm to 6:00 pm and another between 7:00 pm to 8: 00 pm.The observation was carried out for three scenarios simultaneously – base, manually controlled traditional and smart. In the base scenario, the bulb is always in ON state from 5:00 pm to 9:00 pm. The bulb is not controlled automatically or manually and retained in ON state. In the manually controlled scenario, the light switched to OFF condition manually as the obstacle moved away from the place. The smart scenario is one where PARSLAD device assists the light bulb. Detailed description of three scenarios is provided in Table 8. In the same location and under quite similar conditions, the measurement for 0.5 W and 8 W LED bulb was done for the three scenarios as stated before. Table 9 provides the energy spent from 5:00 pm to 9:00 pm for three scenarios with three light bulbs and also shows the annual energy consumption. Annual energy consumption is calculated considering the occurrence of intervention that took place in same manner for 365 days from 5:00 pm to 9:00 pm. In Table 10, the annual cost is calculated for three scenarios in both domestic and commercial use. 

**Figure 5:**
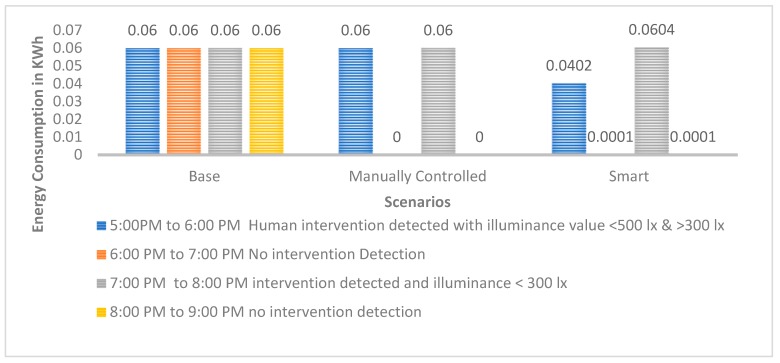
Graph for energy consumption of a 60 W incandescent light bulb in three scenarios.

**Figure 6:**
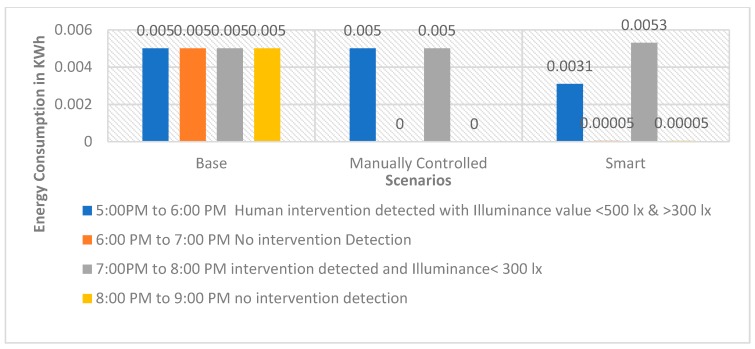
Graph for energy consumption of a 0.5 W LED light bulb in three scenarios.

**Figure 7:**
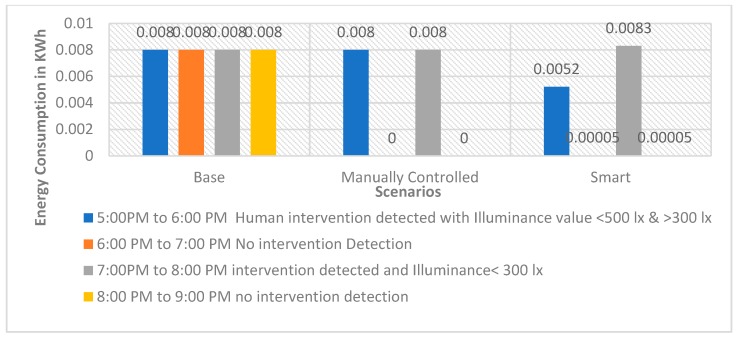
Graph for Energy Consumption of 8 W LED Light Bulb in three scenarios.

Graphs as shown in Figure 5, Figure 6 and Figure 7 for the 60 W incandescent, 0.5 W LED and 8 W light bulb, respectively, for 4 h of observation of the base, manually controlled and smart scenarios. It can be noted that PARSLAD with 60 W incandescent, reduces energy consumption by 58% in contrast to the base scenario and 16% in contrast to the manually controlled scenario, respectively. 

PARSLAD with the 8 W LED bulb gives 57.5% and 15% of energy consumption reduction in comparison to the base and manually controlled scenario, respectively. Thus, the device works efficiently for both incandescent and LED bulbs. PARSLAD with 0.5 W LED reduces energy consumption by 35% in comparison to the base scenario. On comparing the smart and manually controlled scenario, energy consumption shoots up by 0.0003 KWh. Thus, the manually controlled scenario works better than PARSLAD for a 0.5 W LED light bulb. But this is surpassed and PARSLAD provides an improved solution when additional numbers of LED light bulbs are added, as shown in Table 11.

Figure 5, Figure 6 and Figure 7 clearly show the increased energy consumption at 7:00 pm to 8:00 pm when intrusion is perceived in the absence of the prescribed daylight distribution. This upsurge in the smart scenario is due to the power expended for the operation of device along with ON state of light bulb. The energy upshot meant for one hour is adjustable when bearing in mind the reduction of power consumption in the four hours instance of 60 W incandescent and 8 W LED light bulbs. Nevertheless, in the case of a 0.5 W LED bulb, the smart scenario consumes more energy during the monitored four hours. Thus, the number of light bulbs controlled by a PARSLAD device is increased for the purpose of demonstrating the efficiency of the device. 

The readings for three scenarios along with four energy comparison parameters are shown in Table 11 (energy consumption ratio of base scenario contrary to smart scenario (ECR_BS_), Energy consumption ratio of manually controlled scenario counter to smart scenario (ECR_MS_), relative energy saving ratio of base scenario contrary to smart scenario (RESR_BS_) and relative energy saving ratio of manually controlled scenario counter to smart scenario (RESR_MS_)). 

ECR_MS_ can be seen as above 100% and RESR_MS_ as negative for 0.5 W LED light bulb for up to 8 light bulbs indicating a smart scenario for consumption of a larger volume of energy than manually controlled, but it is to be noted that in the manually controlled scenario, the light is in OFF state for two hours whereas, in a smart scenario, the PARSLAD is in ON state during the course of the four hours providing better lighting control and monitoring. The device turns out to be highly resourceful in large indoor and outdoor buildings which deploy many LED bulbs and high wattage consuming bulbs. The device is efficient for 0.5 W LED bulbs which has power consumption smaller than that for zero-watt incandescent light bulbs. Consequently, in LED light bulbs, the device is efficient even for miniscule power consumption commencing with 12 light bulbs. Though the system can be incorporated for both indoor and outdoor environments, slight tailoring of the illuminance threshold values and lighting power sources should be made to support environment-based precision activity. 

### 3.4. Power Analysis of PARSLAD in Alert Mode

The power consumption of PARSLAD with push button in AL mode is tested. It is found that the power consumption is same as in light control mode. As the power consumed by buzzer in ON state is very negligible, the analysis of light control mode and alert mode gives the same result.

## 4. Discussion

### 4.1. Comparison of Three Scenarios

**Figure 8:**
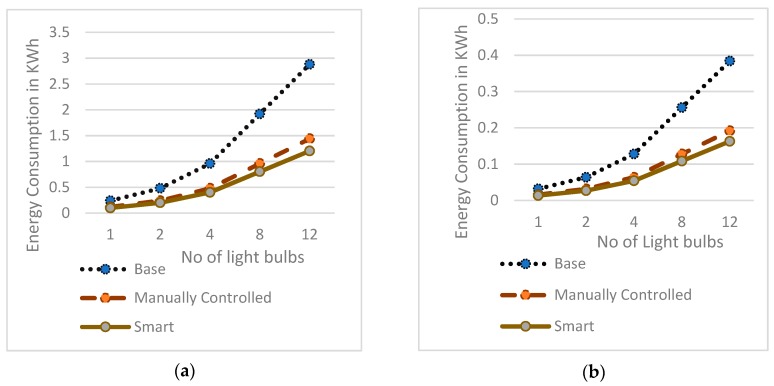

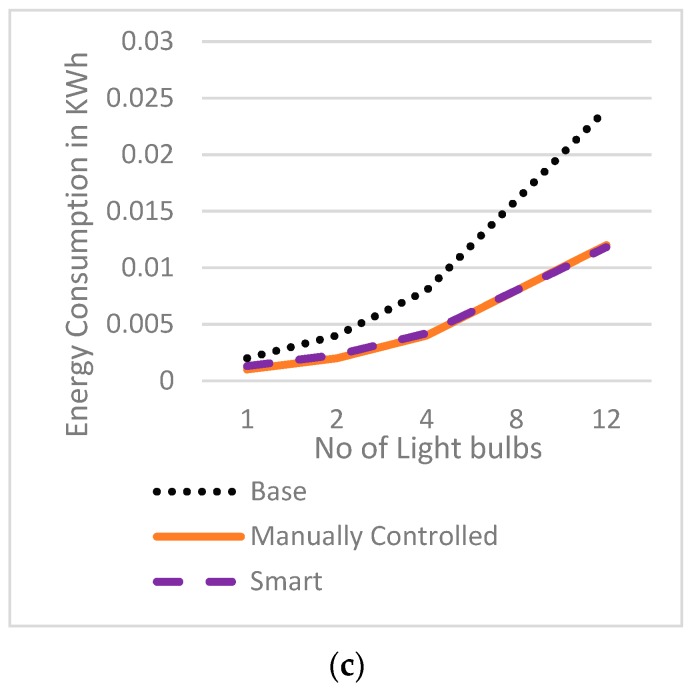
Energy Consumption in KWh vs. number of light bulbs for: (**a**) 60 W incandescent light bulbs in three scenarios, (**b**) 8 W LED light bulbs in three scenarios, (**c**) 0.5 W LED light bulbs in three scenarios.

Details of the power consumptions seen in three scenarios for different numbers of bulbs are given in Figure 8. Figure 8a,b, show the smart scenario of 60 W incandescent and 8 W LED light bulbs as having the lowest consumption in all the cases. Figure 8c shows the smart scenario of a 0.5 W LED light bulb as having less consumption when compared to the base for all the cases, but it also shows a reduction in the energy consumption in the smart scenario for a 0.5 W LED light bulb in comparison to the manually controlled scenario from eight bulbs. Thus, the authors’ proposed model consumes less power for any number of light bulbs for both incandescent and LED light bulbs, but for very low power watt bulbs like 0.5 W, the power is reduced only when it is deployed with eight or more than eight bulbs. For less than eight light bulbs, the consumption of power for smart and manually controlled scenarios was almost the same. As the proposed work can be deployed in any indoor or outdoor lighting application, it is customary to have more than eight LED bulbs if it is only a low wattage for effective use for the low power solution. 

### 4.2. Characteristics o f Energy Consumption Ratios

**Figure 9:**
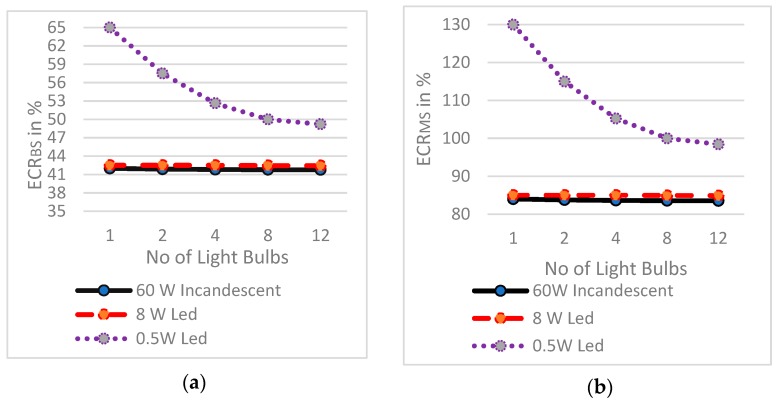
Energy consumption ratio vs. number of light bulbs for: (**a**) Comparison of ECR_BS_ of 60 W incandescent Light, 8 W LED and 0.5 W LED light bulbs and (**b**) comparison of ECR_MS_ of 60 W incandescent Light, 8 W LED and 0.5 W LED light bulbs.

Figure 9 is the graph plotted for the Energy Consumption Ratios of 60 W incandescent, 8 W LED and 0.5 W LED light bulbs. 

Figure 9a,b show a study of the characteristics of ECR_BS_ and ECR_MS_. The study shows the reduction in ratio of energy consumption with increase in the number of light bulbs. It depicts a decrease in ECR_BS_ with an increase in the power of light bulbs and a decrease in ECR_MS_ with decrease in the light bulb power. Consequently, the power consumption of light bulbs is indirectly proportional to ECR_MS_ and ECR_BS_, respectively. ECR_BS_ and ECR_MS_ parameters are scenario- based and it can be seen that as the number of light bulbs increases ECR_BS_ and ECR_MS_ maintain a typical straight line for 60 W incandescent and 8 W LED light bulbs, but they give a quite indirect line for 0.5 W LED light bulb up to eight light bulbs. This is because for up to eight very small power light bulbs, the PARSLAD device alone consumes 50% more power (0.3006 W) than the required power of a low value wattage bulb (0.5 W) to operate in ON state. This problem diminishes by deploying more than eight8 bulbs. We could see the linearity of ECR_BS_ and ECR_MS_ diminishes and an approximate straight line is observed for eight to 12 light bulbs. It can be stated that above 12 number of 0.5 W LED bulbs we observe a straight line similar to the 60 W incandescent and 8 W LED bulb. Thus, the energy consumption ratio parameter is resistant to scalability of light bulbs but in case of very low power bulb, the parameter becomes unaffected only after eight light bulbs.

### 4.3. Characteristics o f Relative Energy Saving Ratios

**Figure 10:**
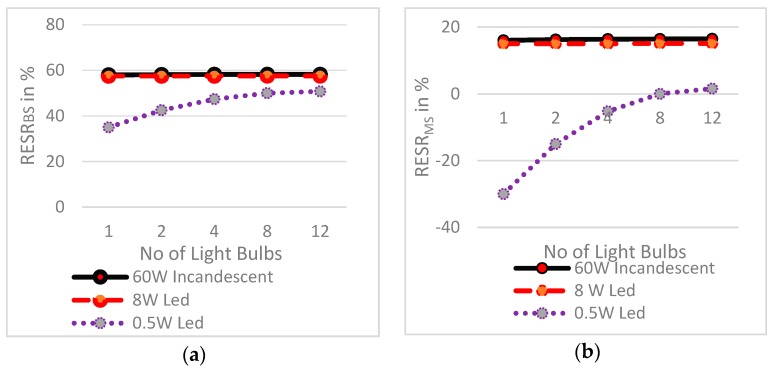
Relative energy saving ratio vs. number of light bulbs for: (**a**) Comparison of RESR_BS_ of 60 W incandescent Light, 8 W LED and 0.5 W LED light bulbs. (**b**) Comparison of RESR_MS_ of 60 W incandescent Light, 8 W LED and 0.5 W LED light bulbs.

Figure 10 shows a plot of the RESR parameters against the number of light bulbs. Figure 10a,b, display the increase in RESR_BS_ and RESR_MS_ with an increase in the number of light bulbs. 

Thus, more energy savings are achieved when the system is deployed with a higher number of light bulbs. It also points out RESR_BS_ and RESR_MS_ are directly proportional to the power of light bulbs. It can also be noted that like the energy consumption ratio parameters, the relative energy saving ratios shows a typical straight line for 60 W incandescent and 8 W LED bulbs. While considering 0.5 W LED bulbs, a low power watt bulb, the RESR parameters varies in a quite logarithmic curve pattern for up to 8 bulbs. Then, the line approaches a straight line like the 60 W incandescent and 8 W LED bulbs.

### 4.4. Energy Savings Graphical Analysis

**Figure 11:**
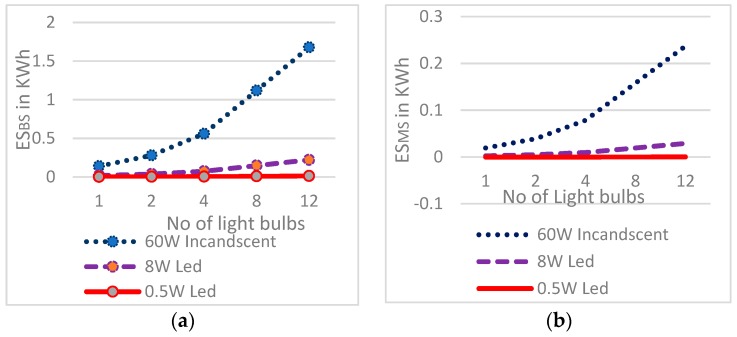
Energy saving ratio vs. number of light bulbs for: (**a**) comparison of ES_BS_ of 60 W incandescent light, 8 W LED and 0.5 W LED light bulbs; (**b**) comparison of ES_MS_ of 60 W incandescent light, 8 W LED and 0.5 W LED light bulbs.

Figure 11a,b depict that the energy savings due to implementation of PARSLAD increases linearly with an increase in the number of light bulbs compared with both the base and manually controlled scenarios, so the energy savings have a linear trend but the scenario-based energy parameters are not only affected by scalability of the light bulbs. Further, the scenario-based energy parameters are linear for a small number of bulbs for very low power. Energy savings of the smart scenario in contrast to the base scenario (ES_BS_) is the difference between the energy consumption of two scenarios, and similarly, for the energy savings of the smart scenario in contrast to the manually controlled scenario (ES_MS_).

### 4.5. Cost Consumption Analysis

The cost analysis for the three scenarios indicates that the smart scenario costs less for all conditions of 60 W incandescent and 8 W LED bulbs. Thus, the proposed method is efficient for both incandescent and LED light bulbs. For 0.5 W, the smart scenario costs more than the manually controlled one for one, two and four light bulbs. The smart and manually controlled scenarios cost the same for eight 0.5 W LED bulbs. The smart scenario starts to show a significant reduction of cost for 12 0.5 W LED bulbs. The cost analysis is detailed in Table 12. The cost of domestic and commercial use is fixed as in Section 3.

## 5. Conclusions

This proposed PARSLAD device, attempts to reduce energy consumption in lights included in the basic utilities as illumination devices. The planned module can work according to the occurrence of intervention and daylight intensity conditions prevailing around the deployed home due to its adaptive nature. This component can equally be used as a prototype for smart light automation from sheds, shelters to royal residences. Hence, the device is pervasive, adaptive and resourceful. The power analysis of PARSLAD proves that the device provides considerable power savings both for incandescent and LED light bulbs. A detailed power analysis of the device is measured. Power consumption of three different light bulbs: 60 W incandescent, 8 W LED and 0.5 W LED bulb for varied scenarios are estimated. The characteristics of scenario-based energy parameters, energy consumption ratio and relative energy saving ratio are detailed. These scenario-based parameters are found to be stable and not affected by the scalability of light bulbs. The problem of exceeding power consumption using the PARSLAD device with very low power (0.5 W) LED bulbs is pointed out. The point to overcome the exceeding power consumption of PARSLAD by deploying it with more than eight light bulbs is depicted clearly. On testing for 800 lumens, higher energy savings for incandescent than LED light bulbs is indicated. Cost consumption analysis also specified that the PARSLAD is efficient for both incandescent and LED light bulbs.

Future studies of device can be carried out in many ways. The main future expansion of the authors is the AL mode of PARSLAD. AL mode can be expanded with GSM facilities for sending alert messages and calls to desired numbers. This mode can also be expanded to facilitate camera to capture image and video for enhanced surveillance. Another upcoming direction is the concept of implementing PARSLAD with a Raspberry Pi system along with a deep learning network to elevate the Arduino-based PARSLAD to an intelligent machine learning-based lighting control and surveillance system (IMLS). Thus, an effectual system could efficiently and simultaneously control the lighting and monitoring in both indoor and outdoor environments. The power consumption of the device can also be reduced further by implementing the device with tier-based activity. In tier-based implementation, PiR and LDR sensing will be carried out in the first tier. On sensing motion, the second tier consisting of light, buzzer and camera will be activated. Currently, the authors are working with the deployment of IMLS. The long time goal of the proposed work is to control all light bulbs (indoor and outdoor) and allow surveillance of a city with a single network technology. The module can be slightly tailored to control more apparatus in the future to automatically satisfy the needs of users with large power savings. 

## Figures and Tables

**Table 1:** Comparison of old devices and the proposed device.

Functionality	Old	Proposed
Dimming	YES	YES
Motion Sensing	YES	YES
Light control with monitoring of environment	NO	YES
Incandescent light support	NO	YES
Compatible for outdoor and indoor environment	ONLY FOR OUTDOOR OR INDOOR. BOTH ARE NOT CONSIDERED IN ONE DESIGN	BOTH
One smart control device for both Incandescent and LED bulb	NO	YES
Problem of automation of low power consuming appliance.	NO	YES

**Table 2:** PARSLAD components.

Components Used	Specifications
RobotDyn light dimmer module	1 channel 3.3 V/5 V logic AC 50Hz, 60 Hz 220 V 110 V, TRIAC-BTA16-600B
Philips LED Bulb	230 V,0.5 W, 20 lumens
Philips Clear GLS Incandescent bulb	230 V,60 W, 800 lumens
Syska LED Lamp	230 V, 8 W, 800 lumens
Light Dependent Resistor	Maximum voltage: 150 Volt DC Maximum wattage: 100 mW Spectral peak: 540 nm Light resistance (10 Lux): 50–100 K Ohm Operating temperature: −30~+70 degree Celsius
Robotix PIR Motion Sensor - HC-SR501	5 V, sensing Distance from few feet – up to 20 feet.
ePro Labs Relay	Four channels, 5 V, each needs 50–60 mA Driver Current
Robo India TECSW Switch Micro - Push to On Button	-
Think-Bots Piezoelectric 3–12 Volt Active Buzzer	5 V

**Table 3:** Comparison of two modes.

Functionality	Light Control (LC)	Alert (AL)
Use of mode	The main purpose is energy saving.	Intrusion monitoring with alarm.
Function of mode	Controls light only when the PiR senses motion.	Controls lights and buzzers when the PiR senses motion.
Indoor application	In indoor applications, when the inhabitants are present.	In indoor application, when the people are away and have locked their homes. Similarly, in closed offices during holidays and a nighttime when employees are not present.
Outdoor application	In outdoor places with frequent human intervention areas like highways, malls, beaches, gardens and parks.	In outdoor places with rare human intervention like high risk places, trespassing into restricted environments, lonely places.
Future Scope of mode	Much more extensive research can enhance the mode to control all appliances (fans, computers, air conditioners).	Can be paired with GSM or a camera facility to send messages or capture images on detection of intervention. This area has wide future scope.
Future method of mode selection	Currently, in this proposed the mode is selected with a push button. In future, neural networks can be incorporated for switching between two modes which can be time-based or event-based, making the system fully automatic.	

**Table 4:** Conditions and logic to be satisfied for PLA.

Perceptive Light Automation Algorithm in Light Control Mode of Operation	
Light State	Condition and Logic to be Satisfied
ON	Intervention detection *AND* Value of Illuminance < 300 lx
DIM	Intervention detection *AND* Value of Illuminance > 300 lx & < 500 lx
OFF	No intervention *OR* Value of Illuminance > 500 lx

**Table 5:** Condition and logic to be satisfied for PLABA.

Perceptive Light Automation with Buzzer Activation Algorithm in Alert (AL) Mode of Operation		
Light State	Buzzer State	Condition and Logic to be Satisfied
ON	ALARM ON	Intervention detection *AND* Value of Illuminance < 300 lx
DIM		Intervention detection *AND* Value of Illuminance > 300 lx & < 500 lx
OFF		Intervention detection AND Value of Illuminance > 500 lx
OFF	ALARM OFF	No intervention *OR* Value of illuminance > 500 lx

**Table 6:** Power analysis of PARSLAD.

S. No	Illuminance (lx)	Condition Tested with Arduino	Voltage (V)	Current (A)	Power (W)	Annual Kilowatt Hour (kWh)	Annual Cost (In Rs)	
							Dom	Com
1	-	Plugging the Arduino to system and uploading the sketch	5.11	0.02	0.1022	0.447636	1.81	3.72
2	>500	0.5 W LED bulb off ^1^	5.13	0.01	0.0513	0.224694	0.91	1.86
3	<500 & >300	0.5 W LED bulb dim ^1^	5.05	0.03	0.1515	0.663701	2.69	5.51
4	<300	0.5 W LED bulb on ^1^	5.01	0.06	0.3006	1.316628	5.33	10.93
5	>500	60 W Incandescent bulb off ^1^	5.14	0.02	0.1028	0.450264	1.82	3.74
6	<500 & >300	60 W Incandescent bulb dim ^1^	5.05	0.04	0.202	0.88476	3.58	7.34
7	<300	60 W Incandescent bulb on ^1^	5.01	0.08	0.4008	1.755504	7.11	14.57
8	>500	8 W LED light bulb off ^1^	5.22	0.01	0.0521	0.228198	0.92	1.89
9	<500 & >300	8 W LED bulb light bulb dim ^1^	5.21	0.03	0.1563	0.684594	2.77	5.68
10	<300	8 W LED bulb light bulb on ^1^	5.20	0.06	0.312	1.368312	5.54	11.36

^1^ Arduino connected to LDR, PiR sensor and relay.

**Table 7:** Power and cost analysis of 60 W incandescent, 8 W LED and 0.5 W LED light bulbs.

Light Bulb State	Description	60 W Incandescent Light Bulb				0.5 W LED Light Bulb				8 W LED Light Bulb			
		Power (W)	Annual Kilowatt hour (KWh)	Annual Cost (Rs)		Power (W)	Annual Kilowatt Hour (KWh)	Annual Cost (Rs)		Power (W)	Annual Kilowatt Hour (KWh)	Annual Cost (Rs)	
				Dom	Com			Dom	Com			Dom	Com
ON	Deprived of PARSLAD	60	262.80	1064.34	2181.24	0.5	2.19	8.87	18.18	8	35.04	141.91	290.83
DIM		Not Applicable in normal operating condition of light bulbs											
OFF		0	0	0	0	0	0	0	0	0	0	0	0
ON	Controlled by PARSLAD	60.4	264.56	1071.45	2195.81	0.80	3.51	14.20	29.11	8.31	36.40	147.42	30.21
DIM		40.2	176.08	713.14	1461.50	0.45	1.98	8.01	16.41	5.16	22.60	91.53	187.58
OFF		0.10	0.45	1.82	3.74	0.0513	0.22	0.91	1.86	0.0521	0.23	0.92	1.89

**Table 8:** Explanation of the three scenarios.

Description	Base Scenario	Manually Controlled Scenario	Smart Scenario
Bulb operation state	ON and OFF.	ON and OFF.	ON, DIM and OFF.
Environment of scenario	The light bulb is always in ON state in the evening and night time. This can be compared to public outdoor environment scenario.	The light bulb is turned to ON state when obstacle reaches and then is switched off. This takes place in indoor environment and in private outdoor environment (e.g.: Terrace, parking areas and garden in homes.)	In indoor, public and private out door environments.
Control of Light bulbs	Centralized control.	Distributed or Decentralized control.	Both Centralized and Decentralized control.
Example of scenario	Currently, the street lights in developing countries are ON even if there is no vehicle or obstacle movement. Emergency Corridors and staircase too have ON Light bulbs.	In home switches turn on the light when needed and then turns OFF manually.	In all types of environment, the light is in ON or DIM state based on the motion sensing and daylight illuminance value.
Advantage and Disadvantage	Though the street light in ON condition throughout in the evening and night time is beneficiary in highways as it is always busy. The criteria is also followed in rare streets in village as the street lights are controlled centrally. This leads to unnecessary power consumption. Even in the case of presence of partial sunlight at sun rising and setting time dim operation is not possible. So light should be either ON or OF	The lights may be in on condition if it is not switched off manually. Example using toilets in night time and forgetting to turn off. Even in the case of presence of partial sunlight at sun rising and setting time dim operation is not possible. So, light should be either ON or OFF.	The light is OFF when there is no intervention. Even in centralized control, outdoor environments, only the lights near the obstacle intervention is in ON or DIM mode and other light bulbs are in OFF state. In case of partial daylight, illuminance light is operated in DIM state on the detection of motion. In indoor environment, if the light is not put off due to forgetfulness of inhabitants the PARSLAD device switches to OFF state. Further this system uses alert control mode to surveillance the nearby areas of light bulbs and alert the surrounding. On future expansion of alert mode with GSM and camera facility will lead to enhanced surveillance and lighting control in one structure.

**Table 9:** Energy consumption in three scenarios.

Light Bulb Specifications	Energy Consumption in KWh			Annual Energy Consumption		
	Base	Manually Controlled	Smart	Base	Manually Controlled	Smart
60 W Incandescent	0.24	0.12	0.1008	87.6	43.8	36.792
8 W LED	0.032	0.016	0.0136	11.68	5.84	4.964
0.5 W LED	0.002	0.001	0.0013	0.73	0.365	0.4745

**Table 10:** Annual cost of three scenarios.

Light Bulb Specifications	Annual Cost in Rupees					
	Base		Manually Controlled		Smart	
	Dom	Com	Dom	Com	Dom	Com
60 W Incandescent	354.78	727.08	177.39	363.54	149.01	305.37
8 W LED	47.30	96.94	23.65	48.47	20.10	41.20
0.5 W LED	2.96	6.06	1.48	3.03	1.92	3.94

**Table 11:** Energy Saving Parameters.

No of Light Bulbs	Light Bulb Specifications	Energy Consumption in KWh			ECR_BS_ (%)	ECR_MS_ (%)	RESR_BS_ (%)	RESR_MS_ (%)
		Base	Manually Controlled	Smart				
1	60 W Incandescent	0.24	0.12	0.1008	42.00	84.00	58.00	16.00
2		0.48	0.24	0.2010	41.88	83.75	58.13	16.25
4		0.96	0.48	0.4014	41.81	83.63	58.19	16.38
8		1.92	0.96	0.8022	41.78	83.56	58.22	16.44
12		2.88	1.44	1.203	41.77	83.54	58.23	16.46
1	8 W LED	0.032	0.016	0.0136	42.50	85.00	57.50	15.00
2		0.064	0.032	0.0272	42.50	85.00	57.50	15.00
4		0.128	0.064	0.0544	42.50	85.00	57.50	15.00
8		0.256	0.128	0.1087	42.46	84.92	57.54	15.08
12		0.384	0.192	0.163	42.45	84.90	57.55	15.10
1	0.5 W LED	0.002	0.001	0.0013	65.00	130.00	35.00	−30.00
2		0.004	0.002	0.0023	57.50	115.00	42.50	−15.00
4		0.008	0.004	0.0421	52.63	105.25	47.38	−5.25
8		0.016	0.008	0.0080	50.00	100.00	50.00	0.00
12		0.024	0.012	0.1181	49.21	98.42	50.79	1.58

**Table 12:** Cost consumption analysis of light bulbs under diverse conditions and scenarios.

Light Specifications	No of Light Bulbs	Base Scenario		Manually Controlled Scenario		Smart Scenario	
		Cost in Rupees		Cost in Rupees		Cost in Rupees	
		Dom	Com	Dom	Com	Dom	Com
60 W Incandescent	1	0.972	1.992	0.486	0.996	0.40824	0.83664
	2	1.944	3.984	0.972	1.992	0.81405	1.6683
	4	3.888	7.968	1.944	3.984	1.62567	3.33162
	8	7.776	15.936	3.888	7.968	3.24891	6.65826
	12	11.664	23.904	5.832	11.952	4.87215	9.9849
8 W LED	1	0.1296	0.2656	0.0648	0.1328	0.05508	0.11288
	2	0.2592	0.5312	0.1296	0.2656	0.11016	0.22576
	4	0.5184	1.0624	0.2592	0.5312	0.22032	0.45152
	8	1.0368	2.1248	0.5184	1.0624	0.440235	0.90221
	12	1.5552	3.1872	0.7776	1.5936	0.66015	1.3529
0.5 W LED	1	0.0081	0.0166	0.00405	0.0083	**0.005265**	**0.01079**
	2	0.0162	0.0332	0.0081	0.0166	**0.009315**	**0.01909**
	4	0.0324	0.0664	0.0162	0.0332	**0.017051**	**0.034943**
	8	0.0648	0.1328	0.0324	0.0664	*0.0324*	*0.0664*
	12	0.0972	0.1992	0.0486	0.0996	***0.047831***	***0.098023***

## Nomenclature

PARSLAD	Pervasive Adaptive Resourceful Smart Lighting and Alerting Device.
PiR	Passive infrared
LC	Light Control
AL	Alert
PLA	Perceptive Light Automation
PLABA	Perceptive Light Automation with Buzzer Activation
LED	Light Emitting Diodes
ZC	Zero Crossing
PWM	Pulse Width Modulation
SYNC	Synchronous
GND	Ground
V_LDR_	Voltage of LDR
IL	Illuminance
ECR	Energy Consumption Ratio
RESR	Relative Energy Saving Ratio
Com	Commercial cost
Dom	Domestic cost
ES	Energy saving
IoT	Internet of Things
LDR	Light Dependent Resistor
BiB	Building -in-Briefcase
IMLS	Intelligent machine learning-based lighting control and surveillance system
ECR_BS_	Energy consumption ratio of base scenario contrary to smart scenario
ECR_MS_	Energy consumption ratio of manually controlled scenario counter to smart scenario
RESR_BS_	Relative energy saving ratio of base scenario contrary to smart scenario
RESR_MS_	Relative energy saving ratio of manually controlled scenario counter to smart scenario
E_B_	Energy in base scenario
E_M_	Energy in manually controlled scenario
E_S_	Energy in smart scenario
ES_BS_	Energy saving of smart scenario in contrast to base scenario.
ES_MS_	Energy saving of smart scenario in contrast to manually controlled scenario.
